# A Strong, Tough and Fire-Retardant Biomimetic Multifunctional Wooden Laminate

**DOI:** 10.3390/polym15204063

**Published:** 2023-10-12

**Authors:** Xiaoshuai Han, Xiaoyi Wang, Wei Tian, Yuli Wang, Jiangbo Wang, Frank Lam, Shaohua Jiang

**Affiliations:** 1 Jiangsu Co-Innovation Center of Efficient Processing and Utilization of Forest Resources, International Innovation Center for Forest Chemicals and Materials, College of Materials Science and Engineering, Nanjing Forestry University, Nanjing 210037, China; 2 School of Materials and Chemical Engineering, Ningbo University of Technology, Ningbo 315211, China; 3 Department of Wood Science, The University of British Columbia (UBC), Vancouver, BC V6T 1Z4, Canada

**Keywords:** wood, biomimetic, mechanical performance, fire-retardant

## Abstract

Mildly delignified wood showed a well-preserved wood cell wall framework, and its derived compressed materials demonstrate excellent mechanical properties and advanced functional material potential. Here, we proposed a simple yet effective approach for making strong, tough, and fire-retardant wooden laminate by a three-step process of mild delignification, infiltrating potassium nonafluoro-1-butanesulfonate (PFBS), and hot-pressing to densify the material. PFBS can be infiltrated into the micro/nano-structures of the mildly delignified wood to achieve a good flame-resistant protective barrier. Flame retardant tests showed that this strong, tough, and fire-retardant wooden laminate has a superior flame-retardant performance to natural wood. Additionally, the wooden laminate also exhibits a simultaneously enhanced tensile strength (175.6 MPa *vs.* 89.9 MPa for natural wood) and toughness (22.9 MJ m^−3^ *vs.* 10.9 MJ m^−3^ for natural wood). Given these attributes, the resulting wooden laminates are identified as promising candidates for high-performance structural applications, fulfilling stringent requirements for both mechanical resilience and flame-retardant efficacy.

## 1. Introduction

Owing to the critical challenges in energy sustainability and ecological preservation, there has been an increasing focus on sustainable materials, such as natural materials and biobased polymers, which have been considered promising candidates that can partly replace petrochemical-based products; this is expected to meet some energy and ecological challenges [1,2,3,4,5]. Wood, as an inherently sustainable natural resource, has garnered significant attention across various domains due to its favorable strength-to-weight ratio, ease of workability, esthetic appearance, and so on [6,7,8,9,10,11,12]. Nonetheless, its widespread application is constrained by inherent limitations, including dimensional instability, suboptimal mechanical properties, and flammability [9,13,14,15,16].

For better use of wood, numerous scientific researchers fabricated some advanced functional wooden materials via wood nanotechnology [17,18]. Their studies have found that wood possesses a unique microstructure where hollow fibers are organized in wood stems in a systematic manner. The typical diameter of wood fibers is about 20–30 μm with a cell wall thickness of 2–8 μm [19,20]. In addition, the wood is composed of various kinds of cell types, such as tracheid cells, pit cells, vessel cells, and so on [21,22]. From the chemical composition aspect, the wood fibers are mainly composed of semi-crystalline cellulose covered by amorphous hemicelluloses and lignin [23,24,25]. This unique hierarchical physical/chemical structure will be beneficial to the functionalization of advanced wooden materials.

Recently, natural strong and tough composites have received a lot of attention. Nacre is an outstanding example; it has a highly ordered “brick-and-mortar” microstructure of 95 vol% calcium carbonate and 5 vol% protein and has superior strength and toughness performance owing to its high concentration of well-aligned nanosheets and strong interface [26,27,28]. Inspired by nature, a lot of strong and tough wood-derived composites were also prepared using either top-down assembly or bottom-up reconstruction methods. Advanced analytical methods have elucidated the inherent mechanisms behind their enhanced properties, which are revealed by modern analysis, mainly including increased density, more hydrogen bonding cross-linking, and filler reinforcement [12,29,30,31,32]. These high-performance wooden composites offer promising applications across a range of sectors, from construction and home furnishings to athletic equipment, the automotive industry, and the aerospace industry [33]. In order to have real applications for strong and tough wooden composites, fire-retardant performance must be given attention. Various techniques exist for enhancing the flame resistance of wood, such as surface coatings, fire retardant additives, and increasing wood density [19,34,35,36]. Consequently, there exists a pressing need for straightforward strategies to develop mechanically robust and fire-retardant bio-inspired wooden composites suitable for real-world applications.

Potassium nonafluoro-1-butanesulfonate (PFBS) is a chemical compound used in the field of fire retardancy. It is known for its effective fire suppression properties and is commonly used as an additive in various fire retardant formulations. One of the key advantages of using PFBS as a fire retardant is its ability to suppress and extinguish fires quickly. It works by interrupting the chemical reactions that occur during a fire, thereby reducing the spread and intensity of the flames. This makes it highly valuable in applications where fast fire extinguishing capabilities are crucial. Additionally, PFBS is known for its high thermal stability, which means it can withstand high temperatures without decomposing or losing its fire-suppressant properties. This makes it suitable for use in demanding environments where extreme heat is a concern. Furthermore, PFBS is chemically stable and has low toxicity, making it a safer option compared to certain traditional fire retardants that can be harmful to humans and the environment. Its non-reactive nature also allows it to be compatible with different materials, making it versatile for use in various industries such as construction, electronics, textiles, and transportation.

Herein, a strong, tough, and fire-retardant biomimetic wooden composite was firstly prepared by chemical pretreatment, vacuum-dipping, and the hot-pressing process. Like the process of nacre growth, the wood cell wall plays the role of the “brick”, and the PFBS plays the role of the “mortar” in the manufacturing process. The obtained wooden composite showed dense macro/microstructure, high mechanical properties, and great fire retardance. The physical, mechanical, and chemical structures were characterized by Fourier transform infrared (FTIR) spectroscopy, X-ray photoelectron spectra (XPS), scanning electron microscopy (SEM), physical and mechanical tests, etc. The strong, tough, and fire-retardant biomimetic wooden laminate demonstrates excellent comprehensive behaviors, showing promising scalable, safe, and structural composite applications.

## 2. Materials and Methods

### 2.1. Materials and Chemicals

Poplar wood was cut into veneer with dimensions of 50 mm × 50 mm × 5 mm (longitudinal × tangential × radial) for use in this study. Sodium chlorite (NaClO_2_, 80%, Alfa Aesar, Haverhill, MA, USA), Acetic acid (HAc, 99.7%, Fisher Chemical, Waltham, MA, USA), and Sulfuric acid (H_2_SO_4_) were purchased from Thermo Fisher Scientific Inc., Shaihai, China. Potassium nonafluoro-1-butanesulfonate (PFBS, 97%), as the flame retardant agent, was purchased from Shanghai Aladdin Biochemical Technology Co., Ltd. (Shanghai, China). All chemicals were used as received without further purification. Deionized (DI) water was used for sample preparation.

### 2.2. Pretreatment for Partial Delignification and Hemicellulose Removal

Poplar specimens underwent a chemical treatment regimen to partially eliminate lignin and hemicellulose. Typically, the wood pieces were bleached three times in an aqueous solution containing 2 wt% NaClO_2_ buffered with HAc at pH 3.5 for 12 h at 70 °C. Subsequent to these treatments, the poplar samples exhibited a whitened appearance and are henceforth designated as pretreated wood (PW). All PW samples were meticulously rinsed with DI water to remove all residual chemicals and decomposed lignocellulosic fragments prior to further utilization.

### 2.3. Vacuum-Dipping and Hot Pressing

The wet pretreated woods were submerged in the prepared PFBS solution (2 wt%) for bulk infiltration under −0.1 MPa for 2 h, thereby ensuring comprehensive penetration of PFBS molecules into the microstructures of the PW. Subsequently, a compressed wood laminate was fabricated by hot-pressing the wet PFBS-immersed pretreated wood under 25 MPa for a period of three days at 105 °C.

### 2.4. Chemical Composition Analysis

The compositional analysis of both natural wood (NW) and PW was conducted in compliance with protocols established by the National Renewable Energy Laboratory protocol [37]. Specifically, a 0.2 g dry powdered sample underwent acidolysis with 3 mL 72 % (*w*/*w*) H_2_SO_4_, and 112 mL DI water was added. Then, the mixture was autoclaved for 60 min at 121 °C, and acid-insoluble lignin was filtered using a medium-porosity 30 mL fritted glass crucible and then oven-dried and weighed. The acid-soluble lignin was determined by UV spectrophotometry (Lambda 950; Perkin, Inc., Waltham, MA, USA) based on the absorbance at 205 nm. The sugar content was analyzed with an Agilent 1200 high-performance liquid chromatography system (Agilent Technologies Inc., Santa Clara, CA, USA). All assays were performed in triplicate.

### 2.5. Fourier Transform Infrared (FTIR) Spectroscopy

The FTIR spectra of samples were obtained using a Fourier transform infrared spectrometer (VERTEX 80 V, Bruker, Bremen, Germany) from 4000 to 400 cm^−1^ at a spectral resolution of 6 cm^−1^ and a total of 32 scans.

### 2.6. X-ray Photoelectron Spectroscopy (XPS)

To investigate the chemical structure of samples, an AXIS UltraDLD X-ray photoelectron spectroscopy was carried out using an Al Kα X-ray source at 225 W at a vacuum pressure of 2 × 10^−8^ torr. The survey scans acquired with a pass energy of 50 eV at 1 eV steps were used to record the low-resolution spectra (0–1200 eV).

### 2.7. X-ray Diffraction (XRD) Analysis

The XRD analysis was carried out using a Rigaku Ultima Ⅳ (Tokyo, Japan) (CuK_α_ radiation with graphite monochromator at 40 kV and 30 mA). The patterns were obtained between 2θ = 5° and 80° with 0.02° steps and a scan speed of 10° min^−1^. The degree of crystallinity index (CI) was determined using the empirical formula reported by Segal et al. [38]:

$$CI(\%)=\frac{I_{200}-I_{am}}{I_{200}}\times 100$$

where *I*_200_ represents the peak at maximum intensity related to the (200) lattice plane, and *I_am_* is the minimum intensity value between the two highest peaks.

### 2.8. Scanning Electron Microscopy (SEM) Analysis

The microstructural characteristics of the NW, PW, and prepared strong, tough, and fire-retardant composite (STFC) were scrutinized via scanning electron microscopy (SEM) (Phenom XL G2, Phenom-World BV, Eindhoven, The Netherlands). Prior to imaging, the specimens were oven-dried and coated with Au by sputter coating before observations to avoid the charging effect. Micrographs were acquired utilizing an accelerating voltage of 1.0 kV and a working distance of 8 mm. 

### 2.9. Mechanical Properties Test

To test the mechanical properties, 50 × 2 mm (L × W) wooden laminate, NW, PW, and STFC were dried at 60 °C for 6 h and then subjected to uniaxial tensile testing using the 3365 universal testing machine (Instron Test Equipment Trading Co., Ltd., Shanghai, China) according to GB/T 1938—2009 [39] The reason for drying at 60 °C was to decrease error induced by moisture content. The specimens were fixed at both ends using a mechanical clamp and stretched along the longitudinal direction to failure. The test was conducted using a 1 kN loader at a 2 mm min^−1^ rate under ambient conditions. In order to avoid slippage at the gripping zone, both ends of the specimen were wrapped using nickel foam during the test. A total of 10 specimens were tested for every sample, and the average and standard deviation were determined.

### 2.10. Flammability Test

The flammability of the NW, compressed pretreated wood (CPW), and STFC (50 mm length × 5 mm width × 2 mm thickness strips and fabricated laminates) were conducted by a butane torch. The samples were horizontally fixed and ignited by a 4 cm butane flame. The exposure duration is contingent upon the flame resistance characteristics of the specimens. Combustion behavior was continually monitored until the sample reached self-sustaining combustion. In addition, the limiting oxygen index (LOI) values were characterized by an LOI tester (FTT0077) in accordance with the GB/T2406.2—2009 [40]. The LOI average value was obtained from three parallel tests.

## 3. Results and Discussion

**Figure 1:**
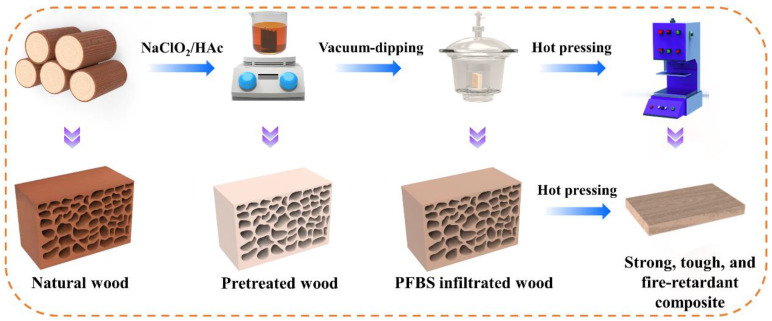
Schematic illustration of the top-down fabrication of strong, tough, and fire-retardant composite by chemical pretreatment, infiltration, and hot-pressing process.

Figure 1 shows the complete process for fabricating strong, tough, and fire-retardant composites. Typically, the composite was fabricated following a three-step process involving (1) pretreatment to remove or break down hydrophobic lignin macromolecules for increasing the porosity of wood and accessibility of cellulose; (2) infiltration to insert fire-retardant reagent (PFBS) into the microstructure of pretreated wood for picking up fire-retardant performance; and (3) hot-pressing of PFBS infiltrated wood to introduce more hydrogen bonding for improved mechanical properties. During the fabrication process, the vacuum-dipping technique can help PFBS molecules enter into the pretreated wood’s microstructure under liquid pressure. The hot-pressing technique is a key step for the densification of wood cell walls, and this packing procedure is similar to nacre formation; therefore, we refer to the product as a biomimetic wooden laminate.

**Figure 2:**
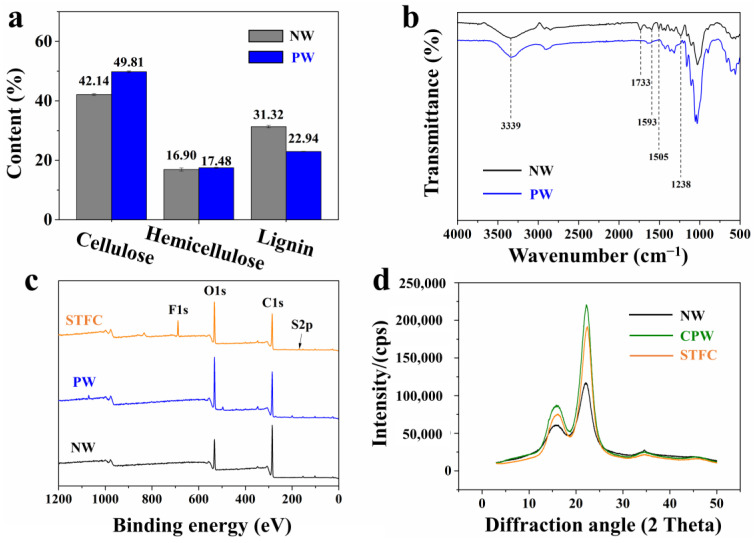
(**a**) The composition of cellulose, hemicellulose, and lignin in natural wood (NW) and pretreated wood (PW). (**b**) FTIR of NW and PW. (**c**) XPS of NW, PW, and strong, tough, and fire-retardant composite (STFC). (**d**) XRD of NW, compressed pretreated wood (CPW), and STFC.

Lignin and parts of hemicelluloses were slightly removed from poplar veneers by structure-retaining delignification in an equal-volume mixture of NaClO_2_ and HAc. There were proportions of 49.81% of cellulose, 17.48% of hemicelluloses, and 22.94% of lignin in PW, suggesting that 26.8% of lignin was removed (Figure 2a). In the delignification process, cellulose is by far less decomposed, and therefore, the cellulosic scaffold in the cell walls remains well. FTIR can be used to obtain an infrared spectrum of absorption or emission of a solid, liquid, or gas, which is helpful in determining the chemical structures of materials [41,42]. As shown in Figure 2b, FTIR spectra also indicated the typical peaks for lignin (1505 cm^−1^ and 1593 cm^−1^ for C=H stretching of the aromatic rings; 1238 cm^−1^ for C-O stretching of the aromatic rings) and hemicelluloses (1733 cm^−1^ for unconjugated carbonyl C=O) were removed by chemical pretreatment (Figure 2b). X-ray photoelectron spectroscopy (XPS) is often used to measure the elemental composition as well as the chemical and electronic state of the atoms of materials [43,44,45]. In this work, to explore changes in the chemical environment of NW, PW, and STFC, XPS spectra were obtained for the three specimens. Figure 2c shows the full-scan spectrum of NW, PW, and STFC. Compared to NW, the O/C ratio of the PW was significantly higher, which also indicated that the lignin was removed from NW. In addition, the existence of F in the STFC indicates that the PFBS was successfully impregnated into the PW. X-ray diffraction (XRD) analysis is an efficient technique to analyze the phase composition, crystal structure, and orientation of materials [46,47,48,49]. As shown in Figure 2d, there is no shift of peak position in NW, CPW, and STFC, indicating compression and modification did not affect the crystalline structure of cellulose. The relative crystallinity of the samples was calculated by the Segal method. The crystalline degree values of the NW, CPW, and STFC were 59.9%, 73.6%, and 72.6%, respectively. The pretreatment process removed amorphous lignin and hemicellulose and preserved the crystal cellulose. Meanwhile, the hot-pressing may induce the crystallization of cellulose in the amorphous region. Moreover, the amorphous PFBS impregnation caused the low crystallinity for STFC compared with CPW. High crystallinity can help improve the mechanical properties of samples, and the excellent mechanical performance of STFC is consistent with the information reflected by the XRD analysis.

**Figure 3:**
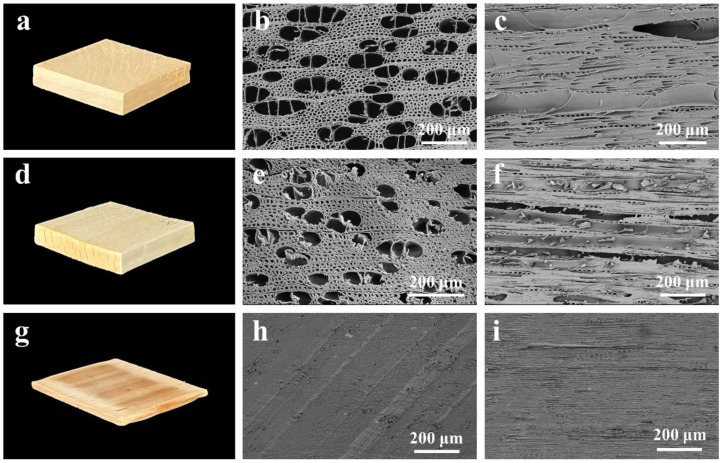
Photographs of (**a**) NW, (**d**) PW, and (**g**) STFC. SEM cross-section images of (**b**) NW, (**e**) PW, and (**h**) STFC. SEM longitudinal-section images of (**c**) NW, (**f**) PW, and (**i**) STFC.

The morphologies of NW, PW, and STFC are shown in Figure 3. Due to delignification, the color of PW looks white compared to NW (Figure 3a,d). The thickness of STFC decreased at the macroscopic level because of the hot-pressing process (Figure 3g). Scanning electron microscopy (SEM) is effective in investigating the surfaces of materials [50,51,52]. In the case of SEM, the transverse section of NW consists of lots of libriform fibers and vessels (Figure 3b). The longitudinal microstructure of libriform fibers and vessels is smooth and clear (Figure 3c). After pretreatment, the connection among wood cells became loosened (Figure 3e), and the longitudinal surface became rough (Figure 3f), indicating some voids appeared in wood cell walls. These voids expose a significant number of cellulose microfibrils, thereby facilitating the infusion of PFBS and subsequent modifications to the cellulosic matrix. Following PFBS impregnation and attainment of equilibrium moisture content, the designated samples underwent hot-pressing at a pressure of 25 MPa and a temperature of 105 °C. Compared to NW and PW, the transverse and longitudinal section images of STFC look very compact and hard. In detail, the wood cell lumen disappeared, and cell walls closely bonded together (Figure 3h). The reason for the compact and hard microstructure can be attributed to two aspects. Firstly, hot-pressing can significantly eliminate various voids, including macropores, mesopores, and micropores, thereby allowing wood fibers to intertwine tightly and pack densely via hydrogen bond formation. Secondly, PFBS serves as a bridging agent for hydrogen bonds between cellulose microfibers during the hot-pressing process. It means the wood fibers play the role of a “brick”, and the PFBS plays a “mortar” function. The above two factors collectively contributed to the formation of the compact structure of STFC, which helps to improve the mechanical properties of samples.

**Figure 4:**
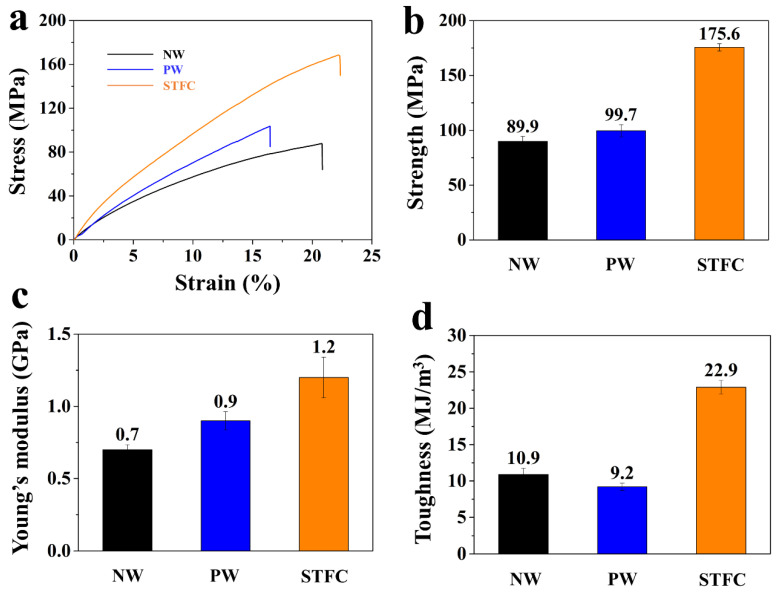
(**a**) Typical tensile stress–strain curves of NW, PW, and STFC. (**b**) The respective tensile strength. (**c**) Young’s modulus and (**d**) toughness values derived from the tensile stress–strain curves.

Tensile stress–strain curves and the corresponding mechanical properties of the NW, PW, and STFC are presented in Figure 4. In the microstructure of wood organization, the lignin plays a role as an adhesive, endowing wood viscoelasticity performance. Owing to lignin depletion, the tensile strain exhibited by the PW was lower than that of the NW. Additionally, the pretreatment process enriched the cellulose content while increasing the exposure of nanocellulose. Nanocellulose possesses superior physical and mechanical properties, including low density (1.6 g cm^−3^), high stiffness (140 GPa), and tensile strength (0.3–1.4 GPa). The PW is mainly composed of nanocellulose, which endows it and its derived STFC with good physical and mechanical performance. In fact, the PW has a better tensile strength (99.7 MPa) than the NW (89.9 MPa) (Figure 4a,b). Due to PBFS infiltration and hot-pressing, the formed STFC showed simultaneously improved tensile strength (175.6 MPa *vs.* 89.9 MPa for NW), Young’s modulus (1.2 GPa *vs.* 0.7 GPa for NW) and toughness (22.9 MJ m^−3^ *vs.* 10.9 MJ m^−3^ for NW). Compared with the mechanical properties of other researchers, we found that the tensile strength is in the middle of the list. Surprisingly, the corresponding toughness is apparently higher than that of most representative studies, such as wooden structural material [53], ultrastrong and tough bulk material [32], etc. In general, strength and toughness are two mutually exclusive properties in materials. The underlying reason for the increased strength and toughness can be explained by three aspects. Firstly, hot-pressing improved the amount of substance per unit volume. Secondly, PBFS infiltration on the surface of cellulose can increase the hydrogen bonding capacity and, therefore, create strong interactions between cellulose nanofibers. Hot-pressing effectively mitigates the steric hindrance between cellulose nanofibers, thereby facilitating their cohesion through hydrogen bonding, using PFBS as a bridging agent. Thirdly, PBFS molecules can be regarded as plasticizers in STFC to increase the fracture strain through steadily forming–breaking–forming hydrogen bonding among nanocellulose under pulling force. The enhancement theory by forming–breaking–forming hydrogen bonding was also demonstrated by Zhou et al. [54]. They report a strategy to leverage secondary bonds (e.g., hydrogen bonds) to produce a printed, recyclable, ultra-strong, and ultra-tough graphite structural material. In their study, they demonstrated how hydrogen bonds between the graphite flakes and NFC play a pivotal role in the superb mechanical performance of the composite.

**Figure 5:**
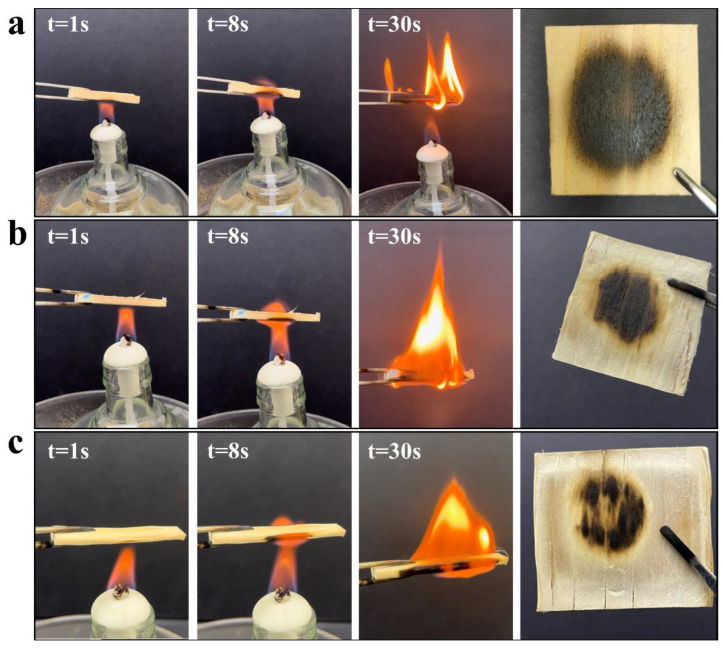
Flame-retardant properties: photographs showing the combustion process of (**a**) NW, (**b**) CPW, and (**c**) STFC.

In order to evaluate the burning behavior of the NW, CPW, and STFC, we exposed the materials to an open alcohol lamp. As shown in Figure 5, natural wood is an extremely flammable material, and it burns almost entirely after 30 s (Figure 5a). It is widely acknowledged that lignin plays an important role as a flame retardant in wood. Research suggested that lignin can only be decomposed at a high temperature by continuous weight loss up to 700–800 °C [55]. Upon its removal, the CPW also exhibited a flammability performance. However, the PW was hot pressed into CPW; given its dense macro- and microstructure, the material became less susceptible to combustion compared to NW (Figure 5b). When PFBS was introduced, the STFC was burning slowly, and the flame was hard to break through in the internal structure (Figure 5c). This observation underscores the effectiveness of PBFS and hot-pressing progress for preventing flame propagation. In addition, the LOI data showed that the NW has a LOI of 22.55%, and the LOI increased to 31.15% for STFC samples, reaching the B1 level of hard-burning material (GB 8624-2012) [56].

## 4. Conclusions

In summary, we successfully fabricated a wood-based material with good physical and mechanical properties and impressive fire-retardant properties by chemical pretreatment, flame-retardant agent infusion, and the hot-pressing process. The FTIR and XPS results revealed that PFBS was successfully grafted into wood fibers via a chemical combination. The morphological observations of the modified wood showed that the wood cells collapsed and merged together due to hot pressing. In addition, the tensile strength, Young’s modulus, and toughness reached 175.6 MPa, 1.2 GPa, and 22.9 MJ m^−3^, respectively, which are greatly superior to those of natural poplar wood. The flame retardancy tests showed that PFBS impregnation modification increased the flame retardancy of poplar wood, thus significantly improving its practical safety. Accordingly, this advanced wooden laminate is suited for diverse applications, including in structural materials, the automotive industry, and so on.

## Data Availability

The data presented in this study are available upon request from the corresponding author.

## References

[1] Zhang M.F., Wang D., Li T., Jiang J., Bai H.Y., Wang S.B., Wang Y., Dong W.F. (2023). Multifunctional Flame-Retardant, Thermal Insulation, and Antimicrobial Wood-Based Composites. Biomacromolecules.

[2] Wang X., Tang S., Wu Z., Fang J., Qin X., Wei L. (2021). Research status of biomass-based composite films with high barrier properties. J. For. Eng..

[3] Mao F., Long L., Zeng G., Chen H., Li Y., Zhou W. (2022). Achieving excellent electromagnetic wave absorption property by constructing VO2 coated biomass carbon heterostructures. Diam. Relat. Mater..

[4] Deng W.-N., Li Y.-H., Xu D.-F., Zhou W., Xiang K.-X., Chen H. (2022). Three-dimensional hierarchically porous nitrogen-doped carbon from water hyacinth as selenium host for high-performance lithium–selenium batteries. Rare Met..

[5] Yang H., Wu F., Zhu G., Li H., Jiang S. (2023). Recent progress of modification and industrialization for nanocellulose towards green building materials. J. For. Eng..

[6] Lainioti G.C., Koukoumtzis V., Andrikopoulos K.S., Tsantaridis L., Ostman B., Voyiatzis G.A., Kallitsis J.K. (2022). Environmentally Friendly Hybrid Organic-Inorganic Halogen-Free Coatings for Wood Fire-Retardant Applications. Polymers.

[7] Zhang Y., Xiao H., Xiong R., Huang C. (2023). Xylan-based ratiometric fluorescence carbon dots composite with delignified wood for highly efficient water purification and photothermal conversion. Sep. Purif. Technol..

[8] Jiang Y.Q., Ru X.L., Che W.B., Jiang Z.H., Chen H.L., Hou J.F., Yu Y.M. (2022). Flexible, mechanically robust and self-extinguishing MXene/wood composite for efficient electromagnetic interference shielding. Compos. Part B Eng..

[9] Yin B., Du W., Zhang Y., Gao X., Ma C., Guo M. (2022). Preparation and photocatalytic performance of biomimetic wood structure cerium vanadate. J. For. Eng..

[10] Gan W.T., Chen C.J., Wang Z.Y., Pei Y., Ping W.W., Xiao S.L., Dai J.Q., Yao Y.G., He S.M., Zhao B.H. (2020). Fire-Resistant Structural Material Enabled by an Anisotropic Thermally Conductive Hexagonal Boron Nitride Coating. Adv. Funct. Mater.

[11] Qu Q., Zhang J., Chen X., Ravanbakhsh H., Tang G., Xiong R., Manshian B.B., Soenen S.J., Sauvage F., Braeckmans K. (2021). Triggered Release from Cellulose Microparticles Inspired by Wood Degradation by Fungi. ACS Sustain. Chem. Eng..

[12] Mi R.Y., Chen C.J., Keplinger T., Pei Y., He S.M., Liu D.P., Li J.G., Dai J.Q., Hitz E., Yang B. (2020). Scalable aesthetic transparent wood for energy efficient buildings. Nat. Commun..

[13] Zhou L.Y., Guo W.Y., Zhang L.R., Che W.B., Yu Y.M. (2022). A top-down strategy for the preparation of flame retardant, robust, and transparent wood-derived films. J. Mater. Res. Technol..

[14] Wang K.H., Meng D., Wang S.H., Sun J., Li H.F., Gu X.Y., Zhang S. (2022). Impregnation of phytic acid into the delignified wood to realize excellent flame retardant. Ind. Crops Prod..

[15] Lu Y., Liang Z., Fu Z., Zhang S. (2022). Research advances and prospect of wood cell wall nanotechnology. J. For. Eng..

[16] Hao X.H., Li M.L., Huang Y.S., Sun Y.H., Zhang K.X., Guo C.G. (2022). High-Strength, Dimensionally Stable, and Flame-Retardant Fast-Growing Poplar Prepared by Ammonium Polyphosphate-Waterborne Epoxy Impregnation. ACS Appl. Polym. Mater..

[17] Jiang F., Li T., Li Y.J., Zhang Y., Gong A., Dai J.Q., Hitz E., Luo W., Hu L.B. (2018). Wood-Based Nanotechnologies toward Sustainability. Adv. Mater..

[18] Chen C.J., Berglund L., Burgert I., Hu L.B. (2021). Wood Nanomaterials and Nanotechnologies. Adv. Mater..

[19] Samanta P., Samanta A., Montanari C., Li Y.Y., Maddalena L., Carosio F., Berglund L.A. (2022). Fire-retardant and transparent wood biocomposite based on commercial thermoset. Compos. Part A Appl. S..

[20] Hoglund M., Johansson M., Sychugov I., Berglund L.A. (2020). Transparent Wood Biocomposites by Fast UV-Curing for Reduced Light-Scattering through Wood/Thiol-ene Interface Design. ACS Appl. Mater. Interfaces.

[21] Wang J.W., Han X.S., Wu W.J., Wang X.Y., Ding L.H., Wang Y.L., Li S.S., Hu J.P., Yang W.S., Zhang C.M. (2023). Oxidation of cellulose molecules toward delignified oxidated hot-pressed wood with improved mechanical properties. Int. J. Biol. Macromol..

[22] Han X.S., Wu W.J., Wang J.W., Tian Z.W., Jiang S.H. (2021). Hydrogen-Bonding-Aided Fabrication of Wood Derived Cellulose Scaffold/Aramid Nanofiber into High-Performance Bulk Material. Materials.

[23] Martinez M.G., Couce A.A., Dupont C., Perez D.D., Thiery S., Meyer X.M., Gourdon C. (2022). Torrefaction of cellulose, hemicelluloses and lignin extracted from woody and agricultural biomass in TGA-GC/MS: Linking production profiles of volatile species to biomass type and macromolecular composition. Ind. Crops Prod..

[24] Terashima N., Kitano K., Kojima M., Yoshida M., Yamamoto H., Westermark U. (2009). Nanostructural assembly of cellulose, hemicellulose, and lignin in the middle layer of secondary wall of ginkgo tracheid. J. Wood Sci..

[25] Kwon S., Zambrano M.C., Pawlak J.J., Venditti R.A. (2021). Effect of lignocellulosic fiber composition on the aquatic biodegradation of wood pulps and the isolated cellulose, hemicellulose and lignin components: Kinetic modelling of the biodegradation process. Cellulose.

[26] Cheng Q.F., Jiang L., Tang Z.Y. (2014). Bioinspired Layered Materials with Superior Mechanical Performance. Acc. Chem. Res..

[27] Zhao H.W., Guo L. (2017). Nacre-Inspired Structural Composites: Performance-Enhancement Strategy and Perspective. Adv. Mater..

[28] Wang L.D., Wang B., Wang Z.Q., Huang J.J., Li K.W., Liu S.P., Lu J.H., Han Z.P., Gao Y., Cai G.F. (2023). Superior Strong and Tough Nacre-Inspired Materials by Interlayer Entanglement. Nano Lett..

[29] Song J.W., Chen C.J., Zhu S.Z., Zhu M.W., Dai J.Q., Ray U., Li Y.J., Kuang Y.D., Li Y.F., Quispe N. (2018). Processing bulk natural wood into a high-performance structural material. Nature.

[30] Ding Y., Pang Z.Q., Lan K., Yao Y., Panzarasa G., Xu L., Lo Ricco M., Rammer D.R., Zhu J.Y., Hu M. (2022). Emerging Engineered Wood for Building Applications. Chem. Rev..

[31] Liu T., Liu Z., Zhou Z.Z., Shi S.Q., Aladejana J.T., Gong S.S., Fang Z., Li J.Z. (2023). A novel sol-gel strategy for constructing wood fibers and aramid nanofiber nanocomposite with strong, tough and recyclable properties. Compos. Sci. Technol..

[32] Han X.S., Ye Y.H., Lam F., Pu J.W., Jiang F. (2019). Hydrogen-bonding-induced assembly of aligned cellulose nanofibers into ultrastrong and tough bulk materials. J. Mater. Chem. A.

[33] Chen C.J., Kuang Y.D., Zhu S.Z., Burgert I., Keplinger T., Gong A., Li T., Berglund L., Eichhorn S.J., Hu L.B. (2020). Structure-property-function relationships of natural and engineered wood. Nat. Rev. Mater..

[34] Winandy J.E. (2001). Thermal degradation of fire-retardant-treated wood: Predicting residual service life. For. Prod. J..

[35] Chu T.Y., Gao Y.X., Yi L., Fan C.G., Yan L., Ding C., Liu C.C., Huang Q., Wang Z.Y. (2022). Highly fire-retardant optical wood enabled by transparent fireproof coatings. Adv. Compos. Hybrid Mater..

[36] Lin C.F., Karlsson O., Das O., Mensah R.A., Mantanis G.I., Jones D., Antzutkin O.N., Forsth M., Sandberg D. (2023). High Leach-Resistant Fire-Retardant Modified Pine Wood (*Pinus sylvestris* L.) by In Situ Phosphorylation and Carbamylation. ACS Omega.

[37] Sluiter A., Hames B., Ruiz R., Scarlata C., Sluiter J., Templeton D., Crocker D. (2008). Determination of structural carbohydrates and lignin in biomass. Lab. Anal. Proced..

[38] Segal L., Creely J.J., Martin A., Conrad C. (1959). An empirical method for estimating the degree of crystallinity of native cellulose using the X-ray diffractometer. Text. Res. J..

[39] (2009). Method of Testing in Tensile Strength Parallel to Grain of Wood.

[40] (2010). Plastics—Determination of Burning Behavior by Oxygen Index—Part 1: Ambient-Temperature Test.

[41] Cui J., Lu T., Li F., Wang Y., Lei J., Ma W., Zou Y., Huang C. (2021). Flexible and transparent composite nanofibre membrane that was fabricated via a “green” electrospinning method for efficient particulate matter 2.5 capture. J. Colloid Interface Sci..

[42] Wu D., Wang D., Ye X., Yuan K., Xie Y., Li B., Huang C., Kuang T., Yu Z., Chen Z. (2020). Fluorescence detection of Escherichia coli on mannose modified ZnTe quantum dots. Chin. Chem. Lett..

[43] Ma W., Ding Y., Li Y., Gao S., Jiang Z., Cui J., Huang C., Fu G. (2021). Durable, self-healing superhydrophobic nanofibrous membrane with self-cleaning ability for highly-efficient oily wastewater purification. J. Membr. Sci..

[44] Deng Y., Lu T., Zhang X., Zeng Z., Tao R., Qu Q., Zhang Y., Zhu M., Xiong R., Huang C. (2022). Multi-hierarchical nanofiber membrane with typical curved-ribbon structure fabricated by green electrospinning for efficient, breathable and sustainable air filtration. J. Membr. Sci..

[45] Lu T., Liang H., Cao W., Deng Y., Qu Q., Ma W., Xiong R., Huang C. (2022). Blow-spun nanofibrous composite Self-cleaning membrane for enhanced purification of oily wastewater. J. Colloid Interface Sci..

[46] Mao F., Fan X., Long L., Li Y., Chen H., Zhou W. (2023). Constructing 3D hierarchical CNTs/VO_2_ composite microspheres with superior electromagnetic absorption performance. Ceram. Int..

[47] Yuan K., Ye X., Liu W., Liu K., Wu D., Zhao W., Qian Z., Li S., Huang C., Yu Z. (2021). Preparation, characterization and antibacterial activity of a novel Zn(II) coordination polymer derived from carboxylic acid. J. Mol. Struct..

[48] Mao F., Long L., Pi W., Li Y., Zhou W. (2022). X-band electromagnetic absorption and mechanical properties of mullite/Ti_3_AlC_2_ composites. Mater. Chem. Phys..

[49] Hua D., Gao S., Zhang M., Ma W., Huang C. (2020). A novel xanthan gum-based conductive hydrogel with excellent mechanical, biocompatible, and self-healing performances. Carbohydr. Polym..

[50] Zhu L., Li Y., Zhao J., Liu J., Lei J., Wang L., Huang C. (2021). A novel green lignosulfonic acid/Nafion composite membrane with reduced cost and enhanced thermal stability. Chem. Commun..

[51] Deng W., Xu Y., Zhang X., Li C., Liu Y., Xiang K., Chen H. (2022). (NH_4_)_2_Co_2_V_10_O_28_·16H_2_O/(NH_4_)_2_V_10_O_25_·8H_2_O heterostructure as cathode for high-performance aqueous Zn-ion batteries. J. Alloys Comp..

[52] Qu Q., Zhang X., Yang A., Wang J., Cheng W., Zhou A., Deng Y., Xiong R., Huang C. (2022). Spatial confinement of multi-enzyme for cascade catalysis in cell-inspired all-aqueous multicompartmental microcapsules. J. Colloid Interface Sci..

[53] Khakalo A., Tanaka A., Korpela A., Orelma H. (2020). Delignification and Ionic Liquid Treatment of Wood toward Multifunctional High-Performance Structural Materials. ACS Appl. Mater. Interfaces.

[54] Zhou Y.B., Chen C.J., Zhu S.Z., Sui C., Wang C., Kuang Y.D., Ray U., Liu D.P., Brozena A., Leiste U.H. (2019). A printed, recyclable, ultra-strong, and ultra-tough graphite structural material. Mater. Today.

[55] El Moustaqim M., El Kaihal A., El Marouani M., Men-La-Yakhaf S., Taibi M., Sebbahi S., El Hajjaji S., Kifani-Sahban F. (2018). Thermal and thermomechanical analyses of lignin. Sustain. Chem. Pharm..

[56] (2013). Classification for Burning Behavior of Building Materials and Products.

